# Adult-Onset Acute Disseminated Encephalomyelitis in Elderly Filipino Patients: A Case Report and Review of the Literature

**DOI:** 10.7759/cureus.92138

**Published:** 2025-09-12

**Authors:** Jon Stewart H Dy, Arlene R Ng, Ron Pilotin

**Affiliations:** 1 Neurosciences, Mapúa University School of Medicine, Makati, PHL; 2 Neurology, St. Luke’s Medical Center, Quezon City, PHL; 3 Neuroradiology, St. Luke’s Medical Center, Quezon City, PHL

**Keywords:** acute disseminated encephalomyelitis, clinical outcomes, diagnostic dilemma, elderly filipino patients, immunosuppression, treatment challenges

## Abstract

Acute disseminated encephalomyelitis (ADEM) is an autoimmune disease characterized by inflammation of the cerebral hemispheres, brainstem, cerebellum, and spinal cord, often preceded by prodromal infections or vaccinations. The mainstay of treatment is immunosuppression with high-dose steroid therapy, intravenous immunoglobulin (IVIg), or plasma exchange (PE). While most pediatric patients improve with treatment, the prognosis in adults is less favorable. Local studies reporting the epidemiology, prevalence, incidence, treatment outcomes, and prognosis of ADEM are scarce. This case series presents two elderly Filipino female patients diagnosed with adult-onset ADEM who were treated with high-dose steroid therapy, IVIg, and PE. However, both patients experienced disease progression and eventually succumbed five months after the onset of illness. This study also highlights the diagnostic dilemma that clinicians may face when diagnosing ADEM in the elderly population, along with the treatment challenges and clinical outcomes in patients with a progressive disease course.

## Introduction

Acute disseminated encephalomyelitis (ADEM) is an immune-mediated demyelinating disease of the CNS characterized clinically by new-onset polyfocal neurologic symptoms and/or encephalopathy, coupled with neuroradiological evidence of multifocal demyelination [1]. It is commonly triggered by prodromal infections and immunizations. It is uncommon in elderly adults and may be mistaken for other neurological diseases, such as cerebrovascular disease and CNS infections [1, 2]. Treatment involves immunosuppressive therapy with high-dose IV glucocorticoids, intravenous immunoglobulin (IVIg), or plasma exchange (PE). In adults, there is a higher frequency of ICU admission, poorer prognosis, and higher mortality [3]. Adult-onset ADEM has been increasingly documented in recent years, but there is a paucity of data regarding its prevalence, incidence, and clinical and treatment outcomes in the elderly population [1, 4-7]. In the Philippines, only acute hemorrhagic leukoencephalitis (AHLE), a rare and severe form of ADEM, has been locally documented in an elderly patient [8].

In our study, we present two cases of adult-onset ADEM in elderly Filipino patients admitted to our institution who eventually succumbed to the illness despite treatment with high-dose IV glucocorticoids, IVIg, and PE. 

## Case presentation

The patients’ representative profiles are shown in Table 1. The median age was 66.5 years (range, 60-73 years), and both were female. Both had a time to nadir of 12 weeks after the onset of illness. Symptoms included decreased level of consciousness, gait instability, motor deficits, and aphasia. MRI lesions were seen in the periventricular, cortical, subcortical, thalamic, and brainstem locations. CSF showed lymphocytic pleocytosis in one patient and mildly elevated protein with absent oligoclonal bands in both patients. Treatment given for the first patient was high-dose IV methylprednisolone, PE, and IVIg, while the second patient received high-dose IV methylprednisolone. Both patients had a poor treatment response, with a fatal outcome five months after the onset of illness.

**Table 1:** Patients’ representative profiles. y.o.: Years old; IVMP: Intravenous methylprednisolone therapy; PE: Plasma exchange; IVIg: Intravenous immunoglobulin; OCB: Oligoclonal bands; mg/dL: Milligrams per deciliter.

Case	Age	Sex	Time to nadir	Symptoms	MRI findings	CSF test	CSF result	Treatment	Clinical course
1	73 y.o.	Female	12 weeks	Drowsiness; gait instability; hemiparesis; aphasia	Periventricular; frontoparietal; temporo-occipital; basal ganglia; lateral medulla	WBC; protein; OCB	0; 85 mg/dL; 0	IVMP; PE; IVIg	Fatal after 5 months of illness
2	60 y.o.	Female	12 weeks	Drowsiness; gait instability; quadriparesis	Periventricular; insula; mesial temporal; basal ganglia; medial thalami; midbrain; pons	WBC; protein; OCB	12; 57.5 mg/dL; 0	IVMP	Fatal after 5 months of illness

Patient #1

**Figure 1:**
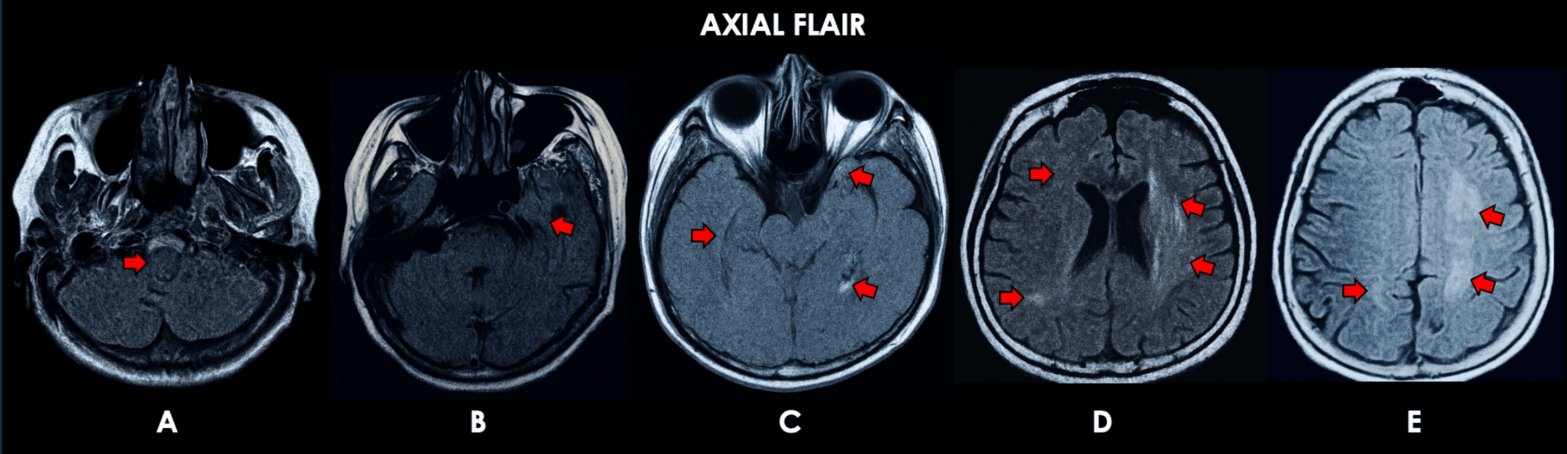
Representative cranial MRI sequences for patient #1. Cranial MRI obtained during the first hospital admission, approximately 2.5 months after the onset of illness. Representative axial brain MRI (FLAIR sequence) images show multiple FLAIR-hyperintense lesions in the right lateral medullary area (A); bilateral temporal and occipital areas (B, C); left frontoparietal area; right frontal and parietal areas; and the left corona radiata and left centrum semiovale (D, E). FLAIR: Fluid-attenuated inversion recovery.

**Figure 2:**
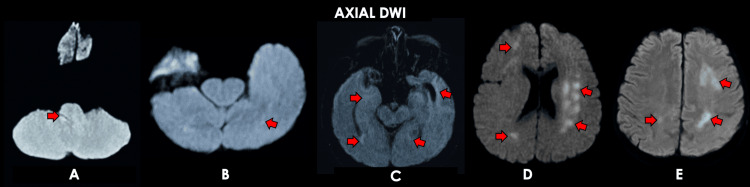
Representative cranial MRI sequences for patient #1. Representative axial brain MRI (DWI sequence) images show restricted diffusion at the sites of the previously described FLAIR-hyperintense lesions in Figure 1, including the right lateral medullary area (A); bilateral temporal and occipital areas (B, C); the left frontoparietal area; right frontal and parietal areas; the left corona radiata; and bilateral centrum semiovale (D, E). FLAIR: Fluid-attenuated inversion recovery; DWI: diffusion-weighted imaging.

The first patient is a 73-year-old Filipino female with controlled hypertension and type 2 diabetes mellitus, independent in all activities of daily living, who presented in October 2021 to another hospital with an acute history of gait instability and generalized weakness. She was initially managed by a neurologist as acute ischemic stroke with antithrombotics (clopidogrel 75 milligrams once daily), piracetam 1,200 milligrams twice daily, and citicoline 1 gram twice daily. Despite initial treatment, there was no improvement, and her symptoms persisted and progressed over two and a half months. Thereafter, she presented with an acute decrease in sensorium, impaired comprehension, word-finding difficulty, and right hemiparesis, for which she was managed by another neurologist at a different hospital as acute ischemic stroke with consideration of a demyelinating lesion, and was then given citicoline 1 g thrice daily, mannitol 0.25 grams per kilogram (g/kg), and cerebrolysin 30 milliliters (215.2 milligrams per milliliter). She was eventually transferred to our institution in December 2021, two months after the onset of her illness. She had no prodromal infections or recent vaccinations and no other known comorbidities. On assessment, she had stable vital signs and was awake and alert, with Broca’s aphasia, right-and-left confusion and finger agnosia; no cranial nerve deficits; flaccid right hemiparesis (best motor strength 2/5); no sensory or cerebellar deficits; no nuchal rigidity; and a positive bilateral toe extensor response. Serological tests revealed microcytic, hypochromic anemia and were negative for serum aquaporin-4 (AQP-4) antibody and myelin oligodendrocyte glycoprotein (MOG) antibody (Table 2). CSF examination showed normal opening and closing pressures; normal cell count and glucose; elevated protein; no evidence of infection; and negative oligoclonal bands, cell block, and cytology (Table 3). Cranial MRI with contrast showed confluent, non-contrast-enhancing, ill-defined T1 hypointense and T2 hyperintense lesions in the right lateral medullary area; bilateral temporal and occipital areas; left frontoparietal area; right frontal and parietal areas; left corona radiata; and left centrum semiovale (Figures 1-2).

**Table 2:** Laboratory tests for patient #1. g/dL: Grams per deciliter; mm³: Cubic millimeters; fL: Femtoliter; mg/dL: Milligrams per deciliter; mmol/L: Millimoles per liter; U/L: Units per liter.

Test	Results	Reference range
Hemoglobin	11.1 g/dL	13.0-17.0 g/dL
Hematocrit	33.30%	40.0-52.0%
White blood cell count	10,300 /mm³	4,800-10,800 /mm³
Neutrophils	68%	40-74%
Lymphocytes	17%	19-48%
Monocytes	13%	3-9%
Platelet count	251,000 /mm³	150,000-400,000 /mm³
Mean corpuscular volume (MCV)	78 fL	82-98 fL
Mean corpuscular hemoglobin (MCH)	26 pg	28-33 pg
Mean corpuscular hemoglobin concentration (MCHC)	32%	32-38%
Creatinine	0.79 mg/dL	0.7-1.3 mg/dL
Blood urea nitrogen (BUN)	11 mg/dL	9-23 mg/dL
Sodium	140 mmol/L	136-145 mmol/L
Potassium	3.8 mmol/L	3.5-5.1 mmol/L
Alanine aminotransferase (ALT)	30 U/L	10-49 U/L
Aspartate aminotransferase (AST)	22 U/L	0-34 U/L
Albumin	3.69 g/dL	3.4-5.4 g/dL
Prothrombin time (control)	11.2 s	11.9-14.2 s
Prothrombin time (test)	12.4 s	11.9-14.2 s
International normalized ratio (INR)	1.05	0.90-1.19
Activated partial thromboplastin time (aPTT)	30 s	29.5-39.9 s
Glycohemoglobin (HbA1c)	6.20%	<5.7%
SARS-CoV-2 PCR	Negative	Negative
Serum aquaporin-4 (AQP4) antibody	Negative	Negative
Serum myelin oligodendrocyte glycoprotein (MOG) antibody	Negative	Negative

**Table 3:** Cerebrospinal fluid analysis for patient #1. cm H₂O: Centimeters of water; mm³: Cubic millimeters; mg/dL: Milligrams per deciliter.

Test	Results	Reference range
Opening pressure	10 cm H₂O	8-18 cm H₂O
Closing pressure	8 cm H₂O	8-18 cm H₂O
Gross description	Clear and free-flowing	Clear and free-flowing
Cells	0 cells/mm³	0-5 cells/mm³
CSF/serum glucose ratio	0.62	0.50-0.60
Protein	85 mg/dL	8-32 mg/dL
Gram stain	Negative	Negative
Bacterial culture and sensitivity	Negative at 3 days	Negative
Japanese encephalitis virus; dengue virus; SARS-CoV-2	Negative	Negative
Acid-fast bacilli (AFB) smear	Negative	Negative
Mycobacterium tuberculosis PCR	Negative	Negative
Mycobacterial culture and sensitivity	Negative at 42 days	Negative
Potassium hydroxide (KOH) prep; India ink	Negative	Negative
Cryptococcal antigen latex agglutination	Negative	Negative
Escherichia coli K1; Haemophilus influenzae; Listeria monocytogenes	Negative	Negative
Neisseria meningitidis; Streptococcus pneumoniae	Negative	Negative
Streptococcus agalactiae; Cryptococcus neoformans	Negative	Negative
Cytomegalovirus; enterovirus; herpes simplex virus 1	Negative	Negative
Herpes simplex virus 2; human herpesvirus 6	Negative	Negative
Human parechovirus; varicella-zoster virus	Negative	Negative
Oligoclonal bands; cell block; cytology	Negative	Negative

Our primary working diagnosis at this time was adult-onset ADEM. She was started on high-dose IV methylprednisolone (1 g daily for five days). Her neurological status improved, now with transcortical motor aphasia, resolution of right-and-left disorientation and finger agnosia, and improvement in motor strength in the right extremities (best motor strength 4/5), and she was discharged after 11 days during the first hospital stay. The interim course showed progressive decrease in sensorium over one month, and she was subsequently readmitted and managed as adult-onset ADEM in relapse. Neurological examination now showed global aphasia, right central facial palsy, flaccid right hemiparesis (best motor strength 2/5), and positive bilateral toe extensor response. Repeat cranial MRI with contrast showed progression of the previously noted confluent T2 hyperintense lesions in similar areas, with no new lesions or areas of enhancement. She was then started on another course of high-dose IV methylprednisolone (1 g daily for five days), immediately followed by five sessions of PE (250 milliliters per kilogram per session), but showed no improvement. She was then given IVIg (2 grams per kilogram over five days) but again showed no improvement, and she eventually succumbed to sepsis from pneumonia after 41 days during the second hospital stay, approximately five months after the onset of her illness.

Patient #2

**Figure 3:**
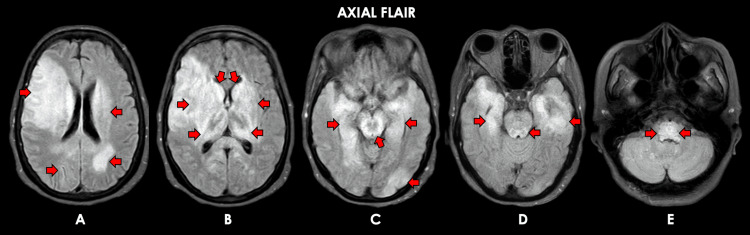
Representative cranial MRI sequences for patient #2. Cranial MRI obtained approximately 2 months after the onset of illness. Representative axial brain MRI (FLAIR sequence) images show confluent, non–contrast-enhancing, ill-defined T2-FLAIR–hyperintense lesions in the bilateral frontoparietal cortical, subcortical, and periventricular regions (A); bilateral insular cortex, caudate nuclei, thalami, and lentiform nuclei (B, C); bilateral medial temporal lobes, midbrain, and pons (D); and bilateral medulla (E). FLAIR: Fluid-attenuated inversion recovery; DWI: Diffusion-weighted imaging.

**Figure 4:**
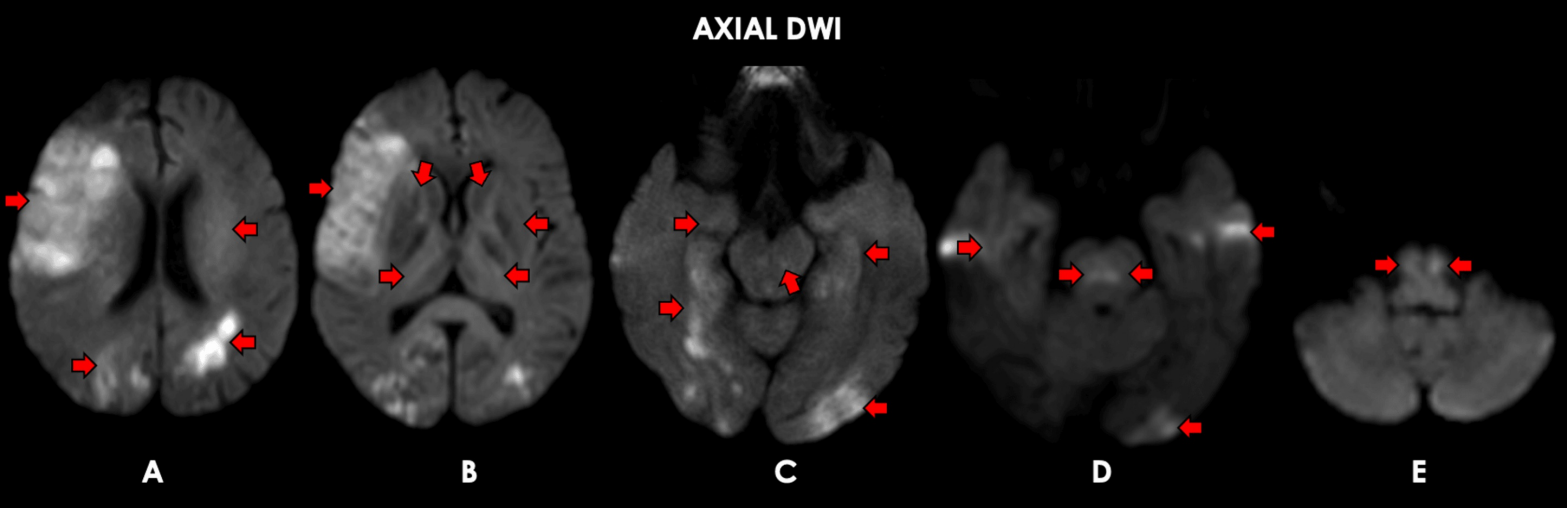
Representative cranial MRI sequences for patient #2. Representative axial brain MRI (DWI sequence) images show restricted diffusion at the sites of the previously described FLAIR-hyperintense lesions in Figure 3, including the bilateral frontoparietal cortical, subcortical, and periventricular regions (A); bilateral insular cortex, caudate nuclei, thalami, and lentiform nuclei (B, C); bilateral medial temporal lobes, midbrain, and pons (D); and bilateral medulla (E). FLAIR: Fluid-attenuated inversion recovery; DWI: Diffusion-weighted imaging.

The second patient is a 60-year-old Filipino female with controlled hypertension and type 2 diabetes mellitus, independent in all activities of daily living, who presented to a different hospital in August 2021 with a five-week history of gait instability, intermittent low-grade fever, generalized weakness, and decreased appetite, for which she was initially managed as a case of enteric fever and given IV ciprofloxacin for 10 days. There was no improvement in her clinical status, and she was again admitted to another hospital in September 2021. During this second hospital admission, she had persistence of her aforementioned symptoms and developed a progressive decrease in sensorium over a span of two weeks, for which she was eventually managed as a case of tuberculous meningoencephalitis and was started on anti-Koch’s therapy, medical decompression, and anticonvulsants (phenytoin and levetiracetam). Her clinical status did not improve despite treatment, and she was eventually transferred to our institution in November 2021. She had neither prodromal infections nor any recent vaccinations, and she had no other known comorbidities. On assessment, she had stable vital signs while on mechanical ventilation but had no eye opening to pain and was unable to follow commands; isocoric pupils 4 mm bilaterally, sluggishly reactive to light; ptosis of the right eye; absent visual threat and corneal reflex in the right eye, with other brainstem reflexes intact; flaccid quadriplegia; generalized hyporeflexia; positive nuchal rigidity; and bilateral toe extensor response. Serological tests revealed peripheral leukocytosis, microcytic, hypochromic anemia, and hyponatremia; negative for serum AQP-4 antibody, MOG antibody, anti-NMDA receptor antibody, and anti-voltage-gated potassium channel antibody (Table 4). Cranial MRI with contrast showed confluent, non-contrast-enhancing, ill-defined T1 hypointense and T2 hyperintense lesions in the bilateral frontoparietal cortical, subcortical, and periventricular regions; bilateral insular cortex; caudate nuclei; thalami and lentiform nuclei; bilateral medial temporal lobes; midbrain and pons; and bilateral medulla (Figures 3-4). A two-hour video EEG showed intermittent generalized theta-delta slowing and triphasic sharp waves, supporting moderate diffuse cerebral dysfunction.

**Table 4:** Laboratory tests for patient #2. g/dL: Grams per deciliter; mm³: Cubic millimeters; fL: Femtoliter; mg/dL: Milligrams per deciliter; mmol/L: Millimoles per liter; U/L: Units per liter.

Test	Results	Reference range
Hemoglobin	12.8 g/dL	13.0-17.0 g/dL
Hematocrit	38.70%	40.0-52.0%
White blood cell count	15,820 /mm³	4,800-10,800 /mm³
Neutrophils	92%	40-74%
Lymphocytes	3%	19-48%
Monocytes	2%	3-9%
Platelet count	374,000 /mm³	150,000-400,000 /mm³
Mean corpuscular volume (MCV)	79 fL	82-98 fL
Mean corpuscular hemoglobin (MCH)	27 pg	28-33 pg
Mean corpuscular hemoglobin concentration (MCHC)	33%	32-38%
Creatinine	0.55 mg/dL	0.7-1.3 mg/dL
Blood urea nitrogen (BUN)	19 mg/dL	9-23 mg/dL
Sodium	133 mmol/L	136-145 mmol/L
Potassium	4.7 mmol/L	3.5-5.1 mmol/L
Alanine aminotransferase (ALT)	55 U/L	10-49 U/L
Aspartate aminotransferase (AST)	25 U/L	0-34 U/L
Albumin	3.69 g/dL	3.4-5.4 g/dL
Prothrombin time (control)	11.2 s	11.9-14.2 s
Prothrombin time (test)	12.4 s	11.9-14.2 s
International normalized ratio (INR)	1.05	0.90-1.19
Activated partial thromboplastin time (aPTT)	29.8 s	29.5-39.9 s
Glycohemoglobin (HbA1c)	6%	<5.7%
SARS-CoV-2 PCR	Negative	Negative
Serum aquaporin-4 (AQP4) antibody	Negative	Negative
Serum myelin oligodendrocyte glycoprotein (MOG) antibody	Negative	Negative
Serum anti-NMDA receptor antibody	Negative	Negative
Serum anti-voltage-gated potassium channel antibody	Negative	Negative

Our primary working diagnosis at this time was to consider adult-onset ADEM, with differentials to rule out tuberculous meningoencephalitis, viral encephalitis, and autoimmune encephalitis. Thereafter, anti-Koch’s therapy and anticonvulsants were continued, and intravenous acyclovir 30 mg/kg/day and meropenem 3 g/day were started. CSF examination showed normal opening and closing pressures, a traumatic tap and lymphocytic pleocytosis, borderline hypoglycorrhachia, mildly elevated protein, no evidence of infection, and negative oligoclonal bands, cell block, and cytology (Table 5). Given the clinical features, laboratory results, and neuroimaging findings, anti-Koch’s therapy, antivirals, and antibiotics were discontinued, and she was started on intravenous pulse methylprednisolone therapy (1 g daily for five days), but with no improvement in her neurologic status. No further immunotherapy was given, as the patient subsequently developed sepsis from multidrug-resistant pneumonia and bacteremia, and she eventually expired after 90 days during the hospital stay, approximately five months after the onset of her illness. 

**Table 5:** Cerebrospinal fluid analysis for patient #2. cm H₂O: Centimeters of water; mm³: Cubic millimeters; mg/dL: Milligrams per deciliter.

Test	Results	Reference range
Opening pressure	13.5 cm H₂O	8-18 cm H₂O
Closing pressure	3.5 cm H₂O	8-18 cm H₂O
Gross description	Clear and free-flowing	Clear and free-flowing
Cells (total)	80 cells/mm³	0-5 cells/mm³
RBCs	68 cells/mm³	0-5 cells/mm³
WBCs	12 cells/mm³	0-5 cells/mm³
Lymphocytes	100%	-
Neutrophils	0%	-
CSF/serum glucose ratio	0.49	0.50-0.60
Protein	57.7 mg/dL	8-32 mg/dL
Gram stain	Negative	Negative
Bacterial culture and sensitivity	Negative at 3 days	Negative
Japanese encephalitis virus; dengue virus; SARS-CoV-2	Negative	Negative
Acid-fast bacilli (AFB) smear	Negative	Negative
Mycobacterium tuberculosis PCR	Negative	Negative
Mycobacterial culture and sensitivity	Negative at 42 days	Negative
Potassium hydroxide (KOH) prep; India ink	Negative	Negative
Cryptococcal antigen latex agglutination	Negative	Negative
Escherichia coli K1; Haemophilus influenzae; Listeria monocytogenes	Negative	Negative
Neisseria meningitidis; Streptococcus pneumoniae	Negative	Negative
Streptococcus agalactiae; Cryptococcus neoformans	Negative	Negative
Cytomegalovirus; enterovirus; herpes simplex virus 1	Negative	Negative
Herpes simplex virus 2; human herpesvirus 6	Negative	Negative
Human parechovirus; varicella-zoster virus	Negative	Negative
Oligoclonal bands; cell block; cytology	Negative	Negative

## Discussion

This case series highlights the complexities of diagnosing and managing ADEM in the elderly population, along with their clinical and treatment outcomes after undergoing high-dose IV methylprednisolone, PE, and IVIg.

Diagnostic challenges

ADEM is an immune-mediated demyelinating CNS disease that occurs most commonly in the pediatric population. In adults, the median age of onset is between 30 and 40 years. In the elderly population, the mean age of onset is 62.3 years [1, 9]. Although a preceding infection or vaccination has been identified in the majority of cases, as many as 40-50% can be idiopathic, especially in elderly adults [9]. Classic ADEM presents as a monophasic demyelinating disease with systemic symptoms including fever, headache, malaise, and myalgias. Neurologic manifestations most commonly include a depressed level of consciousness, unilateral or bilateral long-tract signs, gait abnormalities, brainstem symptoms, and polyfocal deficits. Other less common neurologic symptoms include sphincter dysfunction, cranial nerve palsies, headache, fever, ataxia, vomiting, spinal symptoms, seizures, meningismus, visual loss, extrapyramidal symptoms, and aphasia [9,10]. In elderly patients, fever is less common, while psychiatric symptoms such as visual hallucinations and delusions are more common [9]. Given these clinical features, the diagnosis of ADEM in adults may be mistaken for other neurological diseases such as multiple sclerosis, cerebrovascular disease, posterior reversible encephalopathy syndrome, viral encephalitis, neurosarcoidosis, systemic lupus erythematosus, toxic encephalopathies, adult-onset leukodystrophies, and primary CNS tumors such as lymphoma and glioma [2,3,9]. There are currently no standardized diagnostic criteria for adult-onset ADEM [1,3]. Laboratory and neuroradiological findings supporting the diagnosis of ADEM include peripheral leukocytosis; CSF pleocytosis (51.8%); mildly elevated protein levels (39.1%); negative oligoclonal bands (>80%); EEG findings of diffuse background slowing; and multiple, extensive T2-weighted and fluid-attenuated inversion recovery (FLAIR) white-matter-predominant hyperintense lesions with associated edema and mass effect. Periventricular lesions are the most common location in the elderly; other less frequent locations include the subcortex, basal ganglia, cortex, midbrain, pons, and medulla [1,3,9,10].

Neither patient had a prodromal illness or prior vaccination, but both initially presented with systemic symptoms followed by encephalopathy and multifocal neurological deficits, with a nadir at 12 weeks. Neither case had psychiatric symptoms. Given their age group and the presence of vascular risk factors, both were initially managed as acute cerebrovascular disease, with the second patient subsequently managed as infectious versus autoimmune meningoencephalitis. Laboratory findings for both cases showed peripheral leukocytosis and mildly elevated CSF protein levels, with only one case showing CSF lymphocytic pleocytosis. Neuroradiological findings for both cases showed multiple and confluent T2-weighted and FLAIR hyperintense, non-contrast-enhancing lesions in the periventricular, cortical, subcortical, basal ganglia, and brainstem locations, causing extensive edema and mass effect. The findings in our patients support the characteristic features of ADEM in the elderly population, including the absence of preceding infection or vaccination; similar laboratory and CSF profiles to those seen in younger age groups; EEG findings of diffuse background slowing; and neuroradiological lesions most commonly seen in periventricular locations in supratentorial and infratentorial white matter.

Role of immunosuppressive therapy

Treatment of ADEM includes immunotherapies such as high-dose IV methylprednisolone, PE, and IVIg [3,10]. The use of these therapeutic options is based on the neuroinflammatory pathogenesis of ADEM. To date, none of these therapies has been established in controlled trials. IV methylprednisolone (1 g per day for 3-5 days) is considered the first-line drug and has resulted in full recovery in at least 50% of patients. However, the role of steroids in those presenting late in the disease course is not established [1,3,10]. Poor response to high-dose steroids warrants consideration of plasma exchange. A course of at least four to six plasma exchanges over seven to ten days has been associated with moderate to marked clinical improvement. Small-volume plasma exchanges can be done for those with autonomic dysfunction or those who do not have the resources for conventional plasma exchange. IVIg is an alternative to plasma exchange, but its overall use is limited by high cost and lower quality of evidence [1,3,10].

Our first patient initially received high-dose methylprednisolone with initial improvement, followed by disease progression and another cycle of high-dose methylprednisolone with minimal improvement. This was then followed by PE and IVIg, for which there was again poor treatment response. Our second patient received high-dose methylprednisolone but with no improvement. No further immunosuppressive therapy was given for the second patient because her hospital stay was complicated by sepsis from multiple infections. The findings in our patients show moderate recovery after treatment with high-dose steroid therapy if given early in the course of the disease when the nadir has not been reached. In contrast, there was little to no recovery with high-dose steroid therapy, PE, and IVIg if they were given late in the disease course after the nadir was reached. Given these findings, the most effective therapy for ADEM remains unclear across the elderly population.

Clinical outcomes and prognosis

Complete recovery has been reported in fewer than 50% of patients [3,9]. A less favorable prognosis and higher mortality (10%) are seen in adults with ADEM, with half of deaths occurring more than 3 months after illness onset. Independent predictors of poor outcome include older age, hyperacute onset, severe neurological deficits, prolonged altered mental status, and steroid unresponsiveness [3,8]. Recurrence is also more common in adults (22.6%). Motor deficits, including hemiparesis and ataxia, are the most frequent residual neurological sequelae after recovery [3].

Both patients had rapid progression to a nadir at 12 weeks and a fatal outcome after five months despite immunosuppressive treatment. Predictors of poor outcome in our patients included older age, severe neurological deficits, altered mental status, and steroid unresponsiveness. The findings in our patients are consistent with the literature showing poorer prognosis in the elderly population, especially in those with poor treatment response and severe neurologic deficits as a result of aggressive disease.

Limitations

Our study has several limitations. First, immunosuppressive therapy was initiated late in the disease course, making it difficult to assess the short-term and long-term efficacy had it been initiated earlier. Second, immunosuppressive therapy is available only in major tertiary centers in the Philippines. Lastly, there are no standardized guidelines for the diagnosis and treatment of adult patients with ADEM. Therefore, further studies with larger sample sizes are needed before establishing the diagnostic criteria, safety, efficacy, and treatment outcomes of immunosuppressive therapy in adult-onset ADEM.

## Conclusions

In summary, we present the first local case series of two elderly Filipino patients diagnosed with ADEM who were treated with high-dose steroid therapy, PE, and IVIg. Our study highlights the diagnostic challenges in elderly adults with ADEM that can lead to delays in diagnosis, delayed treatment initiation, and less favorable outcomes. The diagnostic criteria for adult-onset ADEM must be established and standardized to address these issues and to determine its true global prevalence and incidence. Furthermore, in contrast to children and young adults, treatment of ADEM in elderly patients with immunosuppressive therapy remains conjectural, and their clinical outcomes remain unclear. Future studies are needed to evaluate the safety, efficacy, and treatment outcomes of immunosuppressive therapy in elderly adults diagnosed with ADEM.
